# Effect of breastfeeding versus infant formula on iron status of infants with beta thalassemia major

**DOI:** 10.1186/s13006-017-0111-3

**Published:** 2017-04-17

**Authors:** Usama Roshdy El Safy, Manar Mohamed Fathy, Tamer Hasan Hassan, Marwa Zakaria, Mohamed Abdel Kader Al Malky, Mohamed Arafa, Hany El Sayed, Ashgan Al Ghobashy, Boshra Zaho, Attia Abdel Wahab, Mohamed Hosam Mourad

**Affiliations:** 1 Pediatric Department, Faculty of Medicine - Zagazig University, Zagazig, Egypt; 2 Clinical Pathology Department, Faculty of Medicine - Zagazig University, Zagazig, Egypt

**Keywords:** Thalassemia, Breastfeeding, Iron overload

## Abstract

**Background:**

Thalassemia major or Cooley’s anemia is the most severe form of beta thalassemia in which the complete lack of beta protein in the hemoglobin causes a life-threatening anemia requiring regular blood transfusions and extensive ongoing medical care. These extensive, lifelong blood transfusions lead to iron-overload that must be treated with chelation therapy to prevent early death from organ failure. We compared serum iron and ferritin levels amongst infants aged up to one year with beta thalassemia major according to their feeding types, including exclusively breastfed, exclusively formula fed and combined (both breast and formula) fed types.

**Methods:**

Sixty out of 176 screened infants with transfusion dependant beta thalassemia major were recruited from the outpatient clinic of thalassemia at Zagazig University Hospital in Egypt, between 2007 and 2014. Patients were classified into three groups (20 patients per group) according to type of feeding. Group 1: exclusive breastfeeding, around 6–8 feeds per day; group 2: exclusive infant formula feeding, 120–150 ml of formula per kilogram of body weight per day divided into 6–8 feeds and group 3: combined breastfeeding and formula per day.

**Results:**

Serum iron and ferritin levels were lower in group 1 compared to groups 2 and 3. The mean serum iron for group 1 was 73, 87 and 96 ug/dl at 6, 9 and 12 months respectively, while that for group 2 was 85, 99 and 112 ug/dl at 6, 9 and 12 months respectively and for group 3 was 78, 92 and 99 ug/dl at 6, 9 and 12 months respectively. The mean serum ferritin for group 1 was 283, 327 and 497 ng/ml at 6, 9 and 12 months respectively, while that for group 2 was 310, 389 and 591 ng/ml at 6, 9 and 12 months respectively and for group 3 was 291, 345 and 515 ng/ml at 6, 9 and 12 months respectively. The differences were not statistically significant.

**Conclusions:**

Breastfed infants with beta thalassemia major may accumulate less iron than infants fed iron fortified formula anticipating later onset of iron overload in the breastfed infants. Larger studies are needed to support these findings.

## Background

Beta thalassemia is characterized by a reduced production of hemoglobin A (HbA, alpha-2/beta-2), which results from the reduced synthesis of beta-globin chains relative to alpha-globin chains, thus causing an imbalance in globin chain production and hence abnormal erythropoiesis [1]. For clinical purposes, beta thalassemia is divided into thalassemia major (transfusion dependant), thalassemia intermedia (of intermediate severity), and thalassemia minor (asymptomatic, carrier state) [2].

Patients with thalassemia major present in the first year of life with severe anemia; they are unable to maintain a hemoglobin level above 5 gm/dl [3]. For the growing child and the family the advantages of frequent blood transfusions are immediate in terms of wellbeing and improved growth. But this comes at the price of exposure to a potentially toxic iron load, from 7.5 to 15 g per year in a 65 kg individual [4].

Thalassemia is associated with an inappropriate increase in intestinal absorption of iron [5, 6] possibly related to disorders of hepcidin regulation [7], which, when combined with iron derived from transfusion (200–250 mg per unit of blood), leads to an inexorable accumulation of iron in the body tissues. Eventually, extensive iron-induced injury develops in the heart, liver and endocrine organs [8].

The World Health Organization (WHO) and American Academy of Pediatrics (AAP) unequivocally recommend that exclusive breastfeeding is the ideal nutrition for infants and is sufficient to support optimal growth for the first six months of life [9, 10]. Iron concentrations in human milk are low (0.2–0.4 mg/L), but it is thought that the high bioavailability of iron in human milk partly compensates for its low concentrations. The AAP recommends that iron-fortified, cow’s milk-based infant formula is the most appropriate milk feeding from birth to 12 months for normal infants who are not breastfed or who are partially breastfed [11].

We aimed to compare serum iron and ferritin levels between infants with beta thalassemia major on exclusive breastfeeding and those on exclusive formula feeding or combined breast and formula feeding.

## Methods

This comparative study was carried out at a thalassemia outpatient clinic in the pediatric hematology department of Zagazig University Hospital in Egypt, during the period from 2007 to 2014. Sixty out of 176 screened infants with transfusion dependant beta thalassemia major were included. Diagnosis of beta thalassemia was based on medical history and examination, complete blood picture, hemoglobin electrophoresis and DNA analysis for beta globin gene mutations.

According to type of feedings, patients were categorized into three groups. Group 1 included twenty infants (12 males and 8 females) exclusively breastfed* on demand (6–8 feeds per day); group 2 included twenty infants (8 males and 12 females) exclusively formula fed (Standard iron-fortified infant formulas are fortified with approximately 10 to 12 mg of iron, in the form of ferrous sulphate, per quart), 120–150 ml of formula per kilogram of body weight per day divided into 6–8 feeds and group 3 included twenty infants (11 males and 9 females) on combined breast (4–5 feeds/day) and formula milk (2–3 feeds/day).

### Inclusion criteria

1. Transfusion dependant beta thalassemia major
2. Infants up to one year of age
3. No complementary feeding in first year of life

### Exclusion criteria

1. Non transfusion dependant beta thalassemia
2. Other thalassemia syndromes
3. Complementary feeding in first year of life

All thalassemia patients were routinely on calcium, vit D3, L.carnitine and folic acid according to our local standards.

A full history and thorough examination was perfomed on all patients with special emphasis on weight, transfusion data and detailed feeding history, and investigations were performed including blood picture every month and serum iron and serum ferritin every 3 months.

*The definition of exclusive breastfeeding by the WHO implies that the infant receives only breast milk and no other liquids or solids except for drops or syrups consisting of vitamins, mineral supplements, or medicines. Full breastfeeding includes breastfeeding in combination with the supply of water or water-based drinks, including, for example, oral rehydration solutions [9, 10].

### Statistical analysis

The data were checked, entered and analysed using SPSS version 11(SPSS Inc., Chicago, IL, USA). Mean ± standard deviation was used for quantitative variables, and number and percentage were used for qualitative ones. The one-way analysis of variance (ANOVA) is used to compare between groups, whether there are any statistically significant differences between the means of two or more independent (unrelated) groups. *p*-values ≤ 0.05 qualify as significant results and those ≤ 0.001 as highly significant results.

## Results

The mean ages of patients at the time of diagnosis were 6.1, 5.9 and 5.3 months for group 1, 2 and 3 respectively. The three groups were similar as regards age (*p* = 0.653). Also, they were similar concerning socioeconomic status (80 and 20% of families in group 1 were of low and lower middle socioeconomic class respectively versus 75 and 25% in group 2 and 80 and 20% in group 3 respectively, *p* = 0.9). There was no significant difference among the three groups in relation to their weight in the first year (*p* = 0.868, 0.806 and 0.436 at age of 6, 9 and 12 months respectively). There was no significant difference among the three groups as regards pretransfusion hemoglobin level (*p* = 0.934) and number of transfusions (*p* = 0.400). Serum iron and serum ferritin levels were lower in group 1 (exclusive breastfeeding) compared to group 2 and 3. The mean serum iron for group 1 was 73, 87 and 96 ug/dl at 6, 9 and 12 months respectively, while that of group 2 was 85, 99 and 112 ug/dl at 6, 9 and 12 months respectively and group 3 was 78, 92 and 99 ug/dl at 6, 9, 12 months respectively. The mean serum ferritin for group 1 was 283, 327 and 497 ng/ml at 6, 9 and 12 months respectively, while that of group 2 was 310, 389 and 591 ng/ml at 6, 9 and 12 months respectively and group 3 was 291, 345 and 515 ng/ml at 6, 9 and 12 months respectively. The difference among the three groups regarding serum iron and serum ferritin did not reach a statistically significant level (*p* = 0.059, 0.161 and 0.130 for serum iron at 6, 9, 12 months respectively, and *p* = 0.073, 0.070 and 0.080 for serum ferritin at 6, 9, 12 months respectively). Details of our results are listed in Table 1.

**Table 1:** Age, weight, transfusion characteristics, serum iron and serum ferritin in children who were exclusively breastfed, exclusively formula fed, or combination fed

	Exclusively breastfed (*n* = 20)	Exclusively formula fed (*n* = 20)	Combination fed (*n* = 20)	F	P
	Mean ± SD	Mean ± SD	Mean ± SD		
Age at diagnosis (months)	6.1 ± 2.8	5.9 ± 3.1	5.3 ± 2.6	0.430	0.653
Weight (Kg)					
6 months (*n* = 60)	7.2 ± 1.9	6.8 ± 2.5	7.1 ± 2.9	0.142	0.868
9 months (*n* = 60)	8.7 ± 2.5	8.9 ± 1.3	8.4 ± 3.1	0.217	0.806
12 months (*n* = 60)	10.3 ± 3.6	11.1 ± 2.4	9.9 ± 2.8	0.843	0.436
Pretransfusion hemoglobin (gm/dl)	7.9 ± 1.7	7.8 ± 2.1	8 ± 1.2	0.069	0.934
Transfusions/year (n)	5.3 ± 2.6	6.1 ± 1.5	6.0 ± 1.8	0.931	0.400
Serum iron (ug/dl)					
6 months (*n* = 60)	73 ± 13.8	85 ± 18.5	78 ± 14.2	2.969	0.059
9 months (*n* = 60)	87 ± 19.2	99 ± 21.4	92 ± 18.1	1.889	0.161
12 months (*n* = 60)	96 ± 23.8	112 ± 29.7	99 ± 24.6	2.113	0.130
Serum ferritin (ng/ml)					
6 months (*n* = 60)	283 ± 36.9	310 ± 33.4	291 ± 41.7	2.737	0.073
9 months (*n* = 60)	327 ± 81.3	389 ± 92.1	345 ± 82.2	2.799	0.070
12 months (*n* = 60)	497 ± 118.8	591 ± 154.1	515 ± 136.7	2.641	0.080

*SD* standard deviation, *F* analysis of variance

## Discussion

To the best of our knowledge, this is the first study to compare the different feeding modalities in infants with transfusion dependant beta thalassaemia major. In Egypt, there were still new cases of beta thalassaemia major, due to a lack of prevention programs for hemoglobinopathies. Thalassaemia major is normally diagnosed within the first year of life, even being diagnosed in the first few months of life. Early diagnosis is important to begin a treatment program as early as necessary [12].

In our study, the pretransfusion hemoglobin was around 8 gm/dl, which was higher than that reported in the other Egyptian study where the mean pretransfusion hemoglobin level was 5.7 gm/dl [12] and lower than that reported in the multicenter study in Europe and USA where the mean of pretransfusion hemoglobin ranged from 9.11 to 9.88 gm/dl [13].

In healthy infants the mean of serum iron was around 77 mcg/dl and it remained at this level throughout the remainder of the first year [14]. Thalassemia patients absorb too much iron from food due to abnormally low levels of a small peptide, called hepcidin, which regulates iron uptake from the gut. People with thalassemia should produce hepcidin at high levels but instead, these patients have reduced levels of hepcidin [15]. According to researchers at the NIH, iron overload in patients with thalassemia can be due to an overproduction of a protein called GDF15. This suppresses the production of the liver protein, hepcidin, which in turn leads to an increase in the uptake of dietary iron in the gut [16].

This work showed that there was no significant difference among the three groups of patients regarding serum iron, though it was lower in the breastfed group. The wide range in the breast milk iron values reported in the literature may be due, in part, to differences in sampling procedures as well as the stage of lactation. The iron content of human milk is higher in early transitional milk (0.97 mg/ml) but decreases steadily during lactation, reaching a level of approximately 0.35 mg/ml at 1 month of lactation to 0.20 mg/ml at 6 months [17]. The optimal amount of iron in infant formula is debated. For instance, infant formula in Europe typically contains lower amounts of iron than in the United States (approximately 4–7 mg/L compared with 12–13 mg/L) [18]. Factors such as the milk source of iron (e.g. human versus cow), the type of iron compound consumed, the food with which it is eaten, and the iron status of the infant greatly affects iron absorption. For example, greater than 50% of the iron from human milk is absorbed compared with typically less than 12% of the iron from cow milk–derived formula [19].

Additionally, this work showed that there was no significant difference among the three groups of patients concerning serum ferritin, though it was lower in the breastfed group. Serum ferritin is an iron-containing protein complex, found principally in the intestinal mucosa, spleen, and liver, that functions as the primary form of iron stored in the body. Serum ferritin has been reported to correspond to the magnitude of iron stores throughout a wide range. In normal infants and children, we found the concentration of serum ferritin to parallel known changes in iron stores during development. The median serum ferritin concentration was 101 ng/ml at birth, rose to 356 ng/ml at 1 month of age, and then fell rapidly to a median value near 30 ng/ml (95% confidence limits: 7–142 ng/ml) between 6 months and 15 years of age [20]. In patients with thalassemia who are not receiving transfusions, abnormally regulated iron absorption results in increases in body iron burden ranging from 2 to 5 g per year, depending on the severity of erythroid expansion. Regular transfusions may double this rate of iron accumulation [20].

There was no significant difference among the three groups of patients in relation to weight in the first year of life, though it was higher in formula fed group. Clinical presentation of thalassemia major occurs between 6 and 24 months. Affected infants fail to thrive and become progressively pale. Feeding problems, diarrhea, irritability, recurrent fever, and progressive enlargement of the abdomen caused by spleen and liver enlargement may occur. If a regular transfusion program that maintains a minimum hemoglobin concentration of 9.5 to 10.5 g/dl is initiated, growth and development tend to be normal up to 10 to 12 years [21].

There are some limitations in our study including limited number of patients and absence of data regarding their mothers. Limited number of patients were due to the strict eligibility criteria concerning recruitment of babies who did not receive any complementary feeding in their first year of life. Absence of data about mothers of thalassemic babies especially their hemoglobin and iron status can be attributed to their low socioeconomic class that limits their regular follow up during pregnancy.

## Conclusions

Breastfed infants with beta thalassemia major may accumulate less iron than infants feeding on iron fortified formula anticipating later onset of iron overload in the breastfed infants. Larger studies are still needed to support these findings.

## Abbreviations

AAP: American Academy of Pediatrics
ANOVA: Analysis of variance
GDF: Growth differentiation factor
NIH: National Institutes of Health
SD: Standard deviation
WHO: World Health Organization
